# Research on gas-liquid coupled flow field dynamics and atomization characteristics of multi-duct sprayers based on CFD

**DOI:** 10.3389/fpls.2025.1616371

**Published:** 2025-11-20

**Authors:** Jun Li, Hongwei He, Hongtao Fan, Yilin Lie, Yuhao Li, Yachao Cao

**Affiliations:** 1 College of Engineering, South China Agricultural University, Guangzhou, China; 2 Guangdong Laboratory for Lingnan Modern Agriculture, Guangzhou, China; 3 State Key Laboratory of Agricultural Equipment Technology, Beijing, China

**Keywords:** Cfd, multi-duct sprayer, gas-liquid coupling, atomization characteristics, simulation test

## Abstract

This study addresses the issues of prolonged testing cycles and high costs associated with traditional sprayers. Using Computational Fluid Dynamics method, a simulation model of the gas-liquid coupling flow field for multi-duct sprayer was established, and the effects of operational parameters, the air outlet opening angle, interval, and air velocity on droplet deposition and atomization characteristics were systematically investigated. A multi-factor simulation test was conducted by constructing a CFD simulation model, performing multi-polyhedron meshing, and applying the RNG *k*-*ϵ* turbulence model along with the Discrete Phase Model. The results demonstrate that as the flow rate increases from 0.03 kg/s to 0.06 kg/s, the mean thickness of the liquid film and the uniformity index of its distribution both increased, from 197.3 μm and 0.7521 to 340.71 μm and 0.8465 respectively. Medium spray angles and small inner diameter nozzles optimize the uniformity of liquid film distribution, indirectly revealing the effects of each parameter on droplet deposition and its distribution uniformity. When the air outlet opening angle increases from 70° to 80° and then to 90°, the effective working height of the airflow field increases by 0.2 m and 0.1 m, respectively. However, increasing the interval leads to a decrease in the uniformity of the end velocity. The droplets undergo two atomization events within the airflow field. Following the first atomization, the particle size increases due to collisions and merging. The secondary atomization, occurring at a distance of 1.2 m from the air outlet, reduces the particle size and enhances deposition efficiency. Furthermore, as the initial air velocity decreases, the particle size of the droplets within the airflow field tends to increase. The reliability of the CFD simulation model developed in this study were validated through a droplet particle size measurement test. The test results demonstrated that the trend of the measurement values aligned with the simulation values, with the relative error ranging from 11.4% to 15.3%. This research reveals the gas-liquid coupling mechanism within the multi-duct spray flow field, providing a theoretical foundation for the further optimization and modification of this sprayer, thereby significantly reducing costs and improving efficiency.

## 1 Introduction

Pests and diseases represent a critical challenge in fruit cultivation, with their prevention and control being a key component of effective orchard management (Ma et al., 2021; Chen et al., 2023; Xue et al., 2023). Traditionally, chemical control methods have been widely employed due to their effectiveness in managing pests and diseases, as well as their ease of application. However, chemical control methods also present several significant challenges. Chemical residues may persist, leading to environmental pollution, food safety concerns, and potential risks to human health. According to data from the FAOSTAT pesticide use database, global agricultural pesticide consumption reached 3.7 million tons in 2022, marking a 4% increase from 2021 and a 13% rise over the past decade. However, a significant disparity remains in pesticide utilization rates among the world’s leading pesticide-consuming countries. The air-assisted sprayer is one of the most commonly used pieces of equipment in contemporary orchard plant protection systems. Specifically, multi-duct air-assisted spraying technology allows simultaneous application through multiple air ducts. The high-speed airflow generated by the fan causes the leaves to flip, enabling droplets to penetrate the branches and canopy interior, thus enhancing spraying efficiency, uniformity, and overall pesticide application effectiveness (Gu et al., 2014; Zhai et al., 2021). However, the operational effectiveness of the sprayer is constrained by various factors in practical application scenarios (Jiang et al., 2020). Therefore, conducting research on the sprayer using simulation method is crucial (Li et al., 2021). Computational Fluid Dynamics (CFD), the product of integrating computer science and fluid mechanics, has become a vital tool in engineering design and optimization. Simultaneously, CFD has emerged as one of the primary methods for both domestic and international scholars to study the motion characteristics and distribution patterns of the external flow field in orchard air-conveying sprayers (Zhai et al., 2021).

Currently, researchers both domestically and internationally primarily employ CFD simulation method to investigate the airflow characteristics, distribution of airflow fields, and the droplet deposition patterns in orchard air-assisted sprayers (Baetens et al., 2007; Lee et al., 2013; Wei et al., 2023) (García-Ramos et al., 2015). developed several models of air-assisted sprayers utilizing CFD method. These models are capable of simulating the airflow field produced by the sprayer, incorporating critical parameters such as velocity, direction, and turbulence intensity. In order to validate the accuracy of the CFD models, they measured the airflow generated by the sprayer using a 3D acoustic anemometer and compared the results with the CFD simulation outcomes to confirm the model’s reliability (Dekeyser et al., 2013). investigated the airflow characteristics and droplet deposition patterns of the tower-type air-delivered sprayer through both simulation and experimental approaches. The results revealed that droplet deposition on both sides of the tower-type sprayer exhibited asymmetry, and the distribution of the pesticide in the vertical cross-section of the canopy was strongly correlated with the sprayer’s airflow field (Delele et al., 2007). developed a CFD model to simulate and validate a cross-flow air-delivered sprayer. The results indicated that the model effectively predicted droplet dispersion and deposition during the spraying process, thereby offering a robust tool for the design and optimization of the sprayer (Gu et al., 2014) performed an experimental study on the airflow dynamics of the multi-duct sprayer in orchards. The study demonstrated the attenuation of wind speed and jet airflow force as they penetrated the canopy (Endalew et al., 2010). introduced a novel integrated CFD modeling approach and validated it through numerical simulations and wind tunnel experiments for air-assisted spraying in orchards. They also conducted a comprehensive study on the effects of wind speed and direction on spraying performance (Li et al., 2023). assessed critical indicators, including spray performance, droplet size distribution, and spray coverage rate, of the multi-pipe orchard sprayer using field tests and laboratory simulations. They optimized the sprayer’s structure using CFD simulation method to ensure the efficiency of spray airflow and droplet distribution. The findings indicate that the multi-pipeline orchard sprayer can substantially increase spray coverage, reduce pesticide loss, and maintain droplet size uniformity (Huang et al., 2025). used CFD method to model and analyze the airflow field of the wind-driven sprayer in citrus orchards. The study constructed a three-dimensional fluid domain model using SolidWorks, which included the intake zone, rotation zone, diversion zone, and external flow field zone. The numerical simulation was carried out using Fluent software. The gas-phase turbulence was modeled using the RNG *k*-*ϵ* model, and the movement of the droplets was tracked using DPM. The pressure-speed field was coupled based on the SIMPLE algorithm. The model was verified through wind speed boundary experiments. The relative error between the simulated wind speed at the center axis and the measured value was less than 21%, confirming the reliability of the CFD results.; (Duga et al., 2017) developed a 3D CFD model based on computational fluid dynamics principles to simulate the spraying process of air-assisted orchard sprayers. This model accurately predicts and simulates the trajectory of spray drift, reducing drift by at least 50%. The CFD model developed by (Hong et al., 2018) simulates the airflow distribution of the air-assisted sprayer within the tree canopy. By incorporating the sprayer’s movement and the tree canopy’s influence via sliding mesh method and User-Defined Functions (UDFs), the canopy is modeled as a virtual porous medium, thus simplifying the modeling process. The model verification indicates that the simulation results are generally consistent with measured data and can reliably predict airflow distribution. It can provide a reference for improving spray efficiency and predicting spray drift.

In conclusion, both domestic and international researchers have extensively studied the modeling techniques of external flow fields and the airflow distribution of various sprayer types. It has been observed that multi-duct spraying methods significantly enhance the operational performance of sprayers; however, studies addressing the gas-liquid coupling in the external flow field at the multi-duct outlet remain limited. This study aims to investigate the influence of various factors on the external multi-duct flow field of the sprayer during operation in orchard environments. A computational fluid dynamics (CFD) model is employed to establish the external fluid calculation framework for the multi-duct sprayer, with the objective of analyzing the gas-liquid coupling distribution within its external flow field. Based on these findings, the distribution patterns are derived to inform the subsequent optimization of sprayer performance and the refinement of profiling operations.

## 2 Materials and methods

### 2.1 Structure and principle

**Figure 1:**
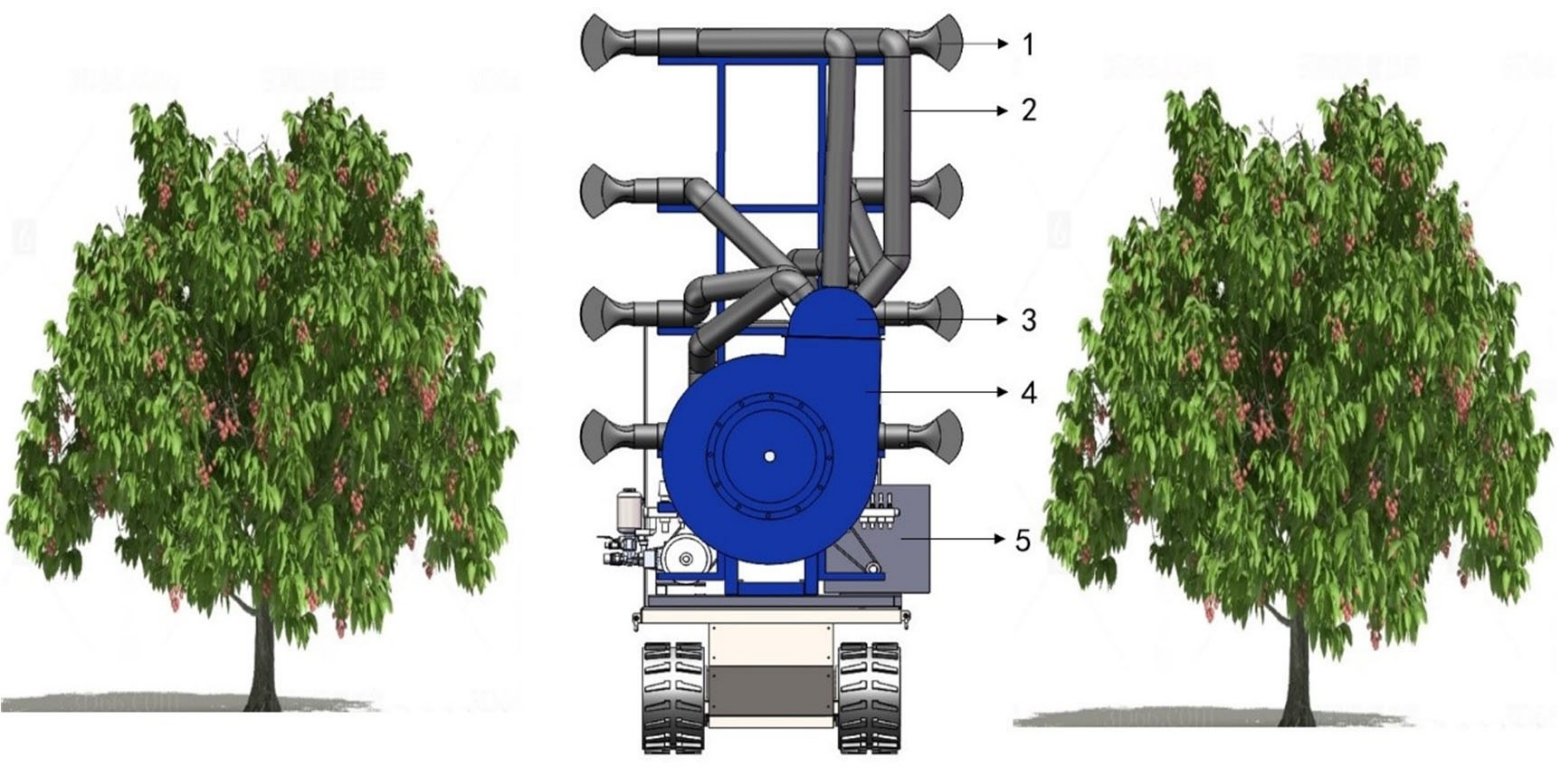
Structure and principle diagram. 1. The air outlet 2. Flexible air duct 3. Eight-outlet air duct distributor 4. Centrifugal fan 5. Gasoline engine.

The air delivery system of the orchard’s multi-duct sprayer consists of a centrifugal fan, an eight-outlet air duct distributor, flexible air ducts, and air outlets, as illustrated in **Figure 1**. When the system operates, the gasoline engine drives the pulley to transmit power to the transmission shaft of the centrifugal fan, which then rotates the fan and generates high-speed airflow. The airflow is directed through the eight-outlet air duct distributor, flows through the flexible air duct, and is finally expelled through the air outlet. After mixing with the atomized liquid droplets sprayed from the atomizing nozzle at the air outlet, the airflow and droplets are directed into the canopies of the fruit trees on both sides (Yang et al., 2024).

### 2.2 Model establishment and meshing

#### 2.2.1 The air outlet structure design

**Figure 2:**
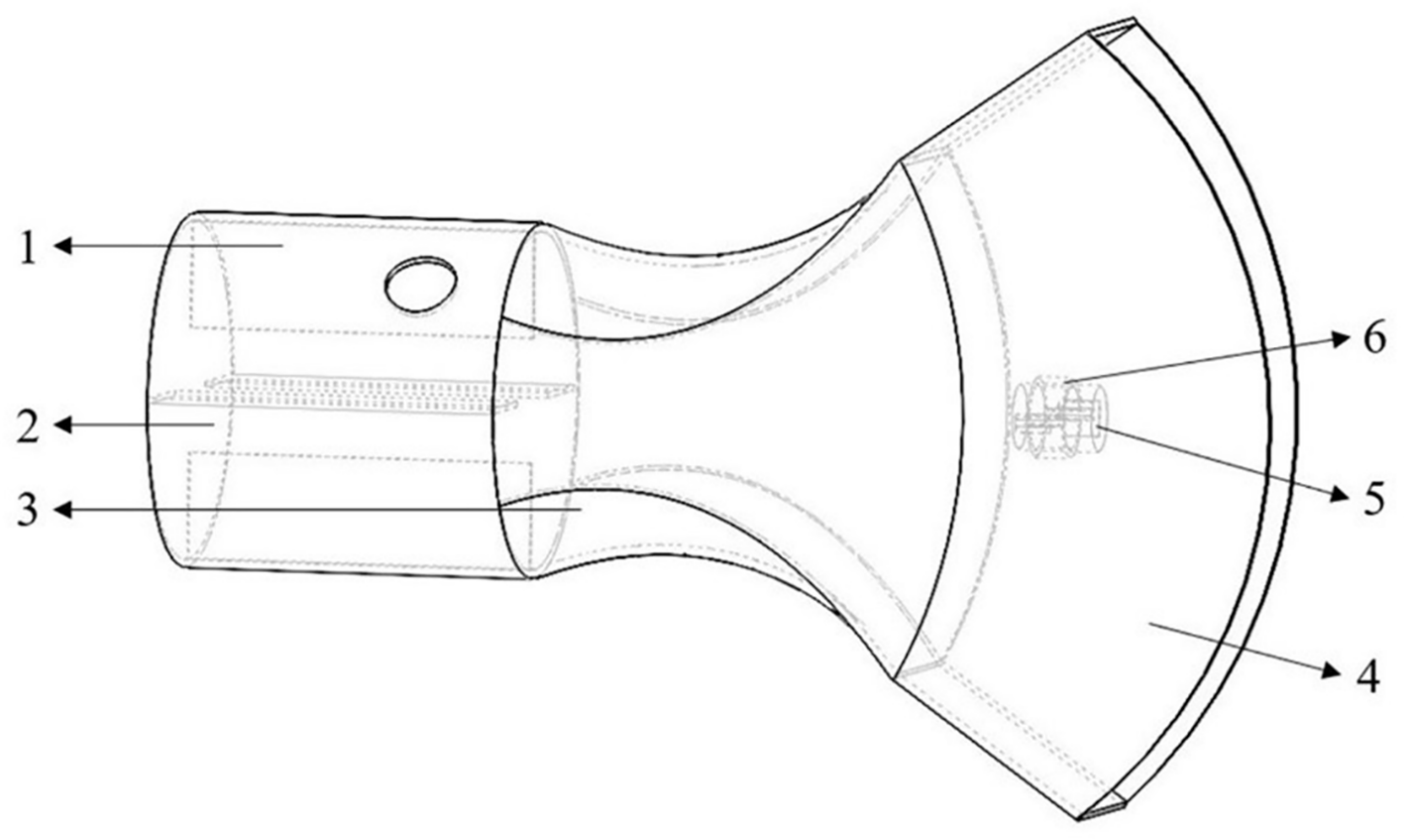
Structure diagram of the air outlet. 1. Deflector plates 2. Cylindrical section 3. A layout section 4. Fan-shaped air outlet section 5. Flat fan atomizer 6. Nozzle fixing frame.

The design of the air outlet structure aims to enhance both the wind speed and the uniformity of the airflow at the outlet. The air outlet consists of a cylindrical section, a layout section, and a fan-shaped section. As shown in **Figure 2** below. The cylindrical section is designed to have a length of 100 mm, the layout section a length of 130 mm, and the fan-shaped section a length of 100 mm. Four deflector plates are incorporated within the cylindrical section. The length of the deflector plates is matched to that of the cylindrical section. This is intended to enhance the energy transfer efficiency of the airflow from the flexible air duct to the outlet and effectively mitigate the energy losses resulting from vortices. The cross-sectional area of the fluid in the layout section first contracts and then expands. In the contraction section, the fluid velocity increases while the pressure decreases. In the expansion section, the velocity continues to increase while the pressure decreases, contributing to the further acceleration of the gas flow. The fan-shaped section serves to augment the vertical flow amplitude while reducing the cross-sectional area of the fluid, thereby maintaining the air velocity at the outlet.

#### 2.2.2 Establishment of multi-duct flow field model

**Figure 3:**
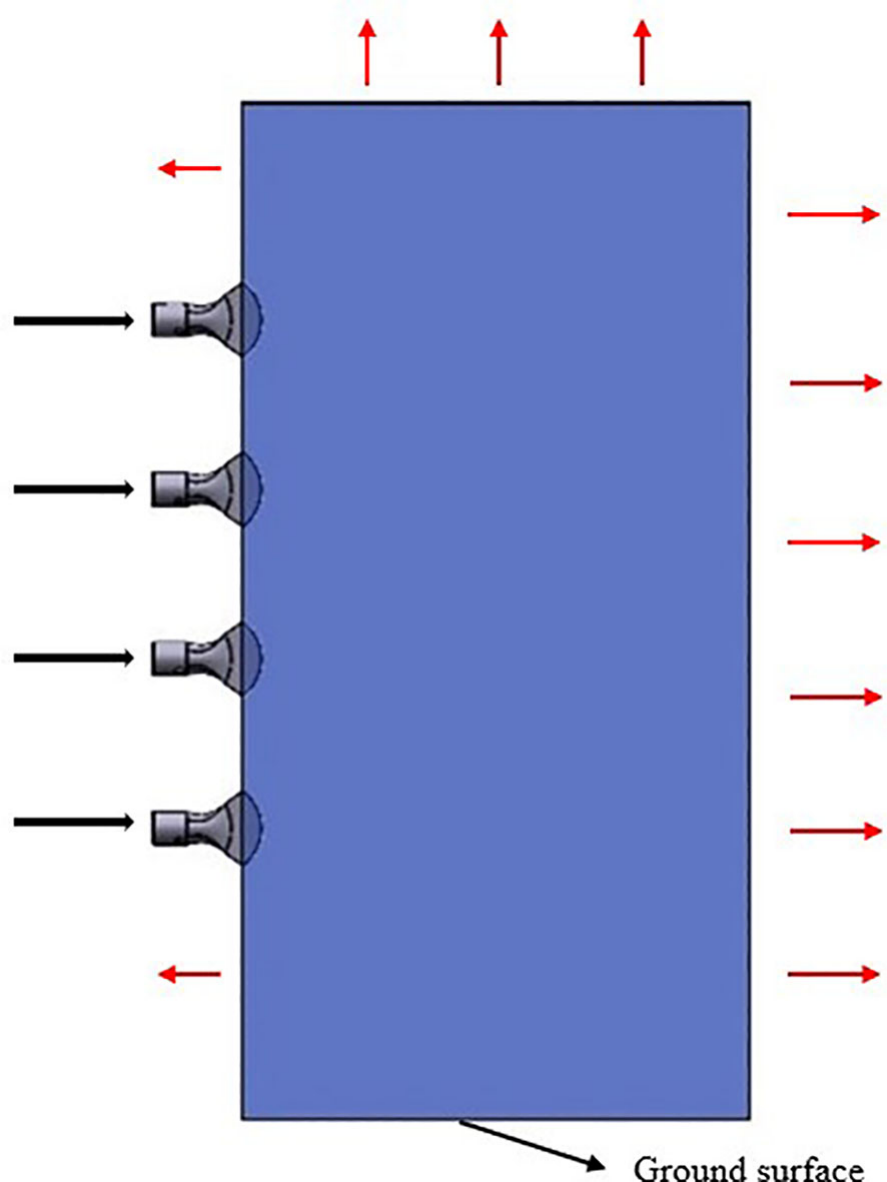
Three-dimensional model of the flow field.

This study aims to investigate the movement characteristics of droplets in the external flow field of a multi-duct sprayer influenced by the airflow field, with the goal of offering insights for its subsequent optimization. Based on the actual working conditions, a geometric model of the outflow field of multiple air ducts was created in SolidWorks software to enhance simulation efficiency and simplify the working area. In this model, the geometry is simplified into a rectangular domain measuring 1500 mm × 400 mm × 3500 mm, which includes four air outlets and a fluid computational domain, as illustrated in **Figure 3**. In the figure, the black arrow represents the entrance of the airflow into the computational domain, and the red arrow represents that the airflow can flow out from here. The area below the computational domain is regarded as the ground surface, and the airflow cannot escape from this area.

The established model is imported into SpaceClaim software, where the fluid computational domain is extracted, and boundary conditions are defined. The bottom of the model is considered as the ground surface. In practical applications, airflow in contact with the ground surface will not escape. Consequently, the bottom boundary is defined as a no-slip wall. Additionally, the wall at the end of the computational domain is defined as a solid boundary to facilitate subsequent analysis of the liquid film thickness. The remaining surfaces of the model, excluding the four air outlets, are considered as surfaces where airflow can escape, and are defined as pressure outlets. Subsequently, the outer surfaces of the four air outlets are defined as walls. Finally, the fluid computational domain is extracted, and the defined model is imported into Ansys Fluent for further processing (Zhang et al., 2022).

#### 2.2.3 Meshing

**Figure 4:**
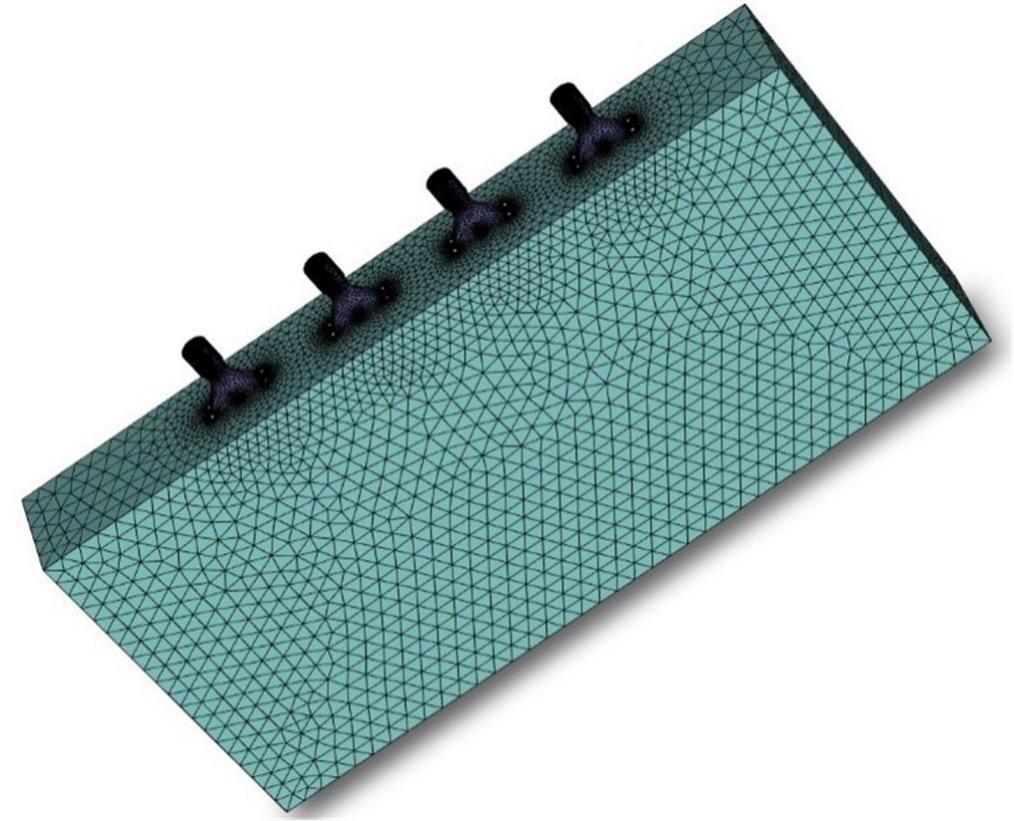
Fluid computational domain grid diagram.

Given that the research model in this study incorporates the multi-channel flow field of droplet movement, the entire model is partitioned into three distinct regions for meshing. The fluid computational domain at the air inlet, as well as the region adjacent to it, undergoes grid refinement, with similar grid refinement applied at the nozzle particle outlet and its vicinity. The remaining fluid domain is discretized using polyhedral grids. Polyhedral grids not only ensure computational accuracy but also reduce computational costs, while offering high grid division efficiency. This study has validated grid independence and identified that the calculation achieves maximum efficiency when the number of grids is 1149854. The grid diagram of the fluid calculation domain is shown in **Figure 4**.

### 2.3 Gas-liquid coupling numerical model

#### 2.3.1 Turbulence model

The airflow field simulated in this study corresponds to a physical field that adheres to turbulent flow characteristics. Currently, the *k-ϵ* model within the Reynolds-averaged Navier-Stokes (RANS) framework is the most commonly employed for simulating gas turbulence. In the Fluent software, the most commonly utilized *k-ϵ* models include the Standard, Realizable, and RNG models. The Standard *k-ϵ* model is the most straightforward and fundamental turbulence model among the two-equation models. It determines the turbulence length and timescales by solving two independent transport equations. During the derivation of this model, it is assumed that the flow is fully turbulent, and the effect of molecular viscosity is negligible. As a result, the Standard model is only applicable in fully turbulent conditions. The Realizable *k-ϵ* model is primarily employed to address scenarios involving particularly high strain rates in turbulent flow. The RNG *k-ϵ* model accounts for the influence of vortices on turbulence, building upon the Standard *k-ϵ* model, and demonstrates superior accuracy and reliability in airflow simulations. Accordingly, this study adopts the RNG *k-ϵ* model and employs its governing equations. The expressions for the transport equations are as follows:

(1) $$\frac{\partial (\rho k)}{\partial t}+\frac{\partial (\rho ku_{i})}{\partial x_{i}}=\frac{\partial }{\partial x_{j}}(\alpha_{k}\mu_{eff}\frac{\partial k}{\partial x_{j}})+G_{k}+G_{b}-\rho \epsilon -Y_{M}+S_{k}$$

(2) $$\frac{\partial (\rho \epsilon )}{\partial t}+\frac{\partial (\rho \epsilon u_{i})}{\partial x_{i}}=\frac{\partial }{\partial x_{i}}(\alpha_{\epsilon }\mu_{eff}\frac{\partial \epsilon }{\partial x_{j}})+C_{1\epsilon }\frac{\epsilon }{k}(G_{k}+C_{3\epsilon }G_{b})-C_{2\epsilon }\rho \frac{\epsilon^{2}}{k}-R_{\epsilon }+S_{\epsilon }$$

In Equations 1, 2, *x* represents the spatial coordinate, *u* is the component of the velocity vector, *k* represents the turbulent kinetic energy, *ϵ* denotes the dissipation rate, *α*_k_ and *α*_ϵ_ are respectively the inverse turbulent Prandtl numbers of *k* and *ϵ*, *μ_eff_* represents the effective viscosity, *G_k_* corresponds to the turbulent kinetic energy generated by the average velocity gradient, *G_b_* is the turbulent kinetic energy generated by buoyancy, *Y_M_* represents the contribution of wave expansion to the total dissipation in compressible turbulence, *C_1_ϵ*, *C_2_ϵ* and *C_3_ϵ* are constants, while *S_k_* and *Sϵ* are user-defined source terms.

#### 2.3.2 Droplet motion model

In computational fluid dynamics (CFD) studies of spray fields, the Discrete Phase Model (DPM) serves as a crucial method for studying particle motion. It has been extensively validated in the literature and has been shown to effectively simulate particle movement within spray fields (Adeniyi et al., 2017). In DPM simulations, the airflow, representing the continuous phase, is iteratively solved using the Euler equation, while droplet particles, representing the discrete phase, are iteratively computed using the Lagrange equation. Hence, this model is referred to as the Euler-Lagrange model, and its governing transport equation is presented in Equation 3 as follows:

(3) dvpdt=18μCDRe24ρPdp2(v−vp)+g(ρp−ρ)ρp

In the formula, *v* represents the velocity of the continuous phase (m/s); *v_p_* represents the discrete phase velocity (m/s); *μ* represents dynamic viscosity (Pa·s);*C_D_* refers to the drag coefficient, *Re* indicates the relative Reynolds number, *ρ_p_* denotes the particle density (kg/m³), *ρ* represents the gas density (kg/m^3^), *d_p_* represents the particle diameter (m), and *g* represents the gravitational acceleration (m/s²).

The drag coefficient is typically associated with the Reynolds number. In the Discrete Phase Model (DPM), particles are assumed to be spherical by default. The expression for the drag coefficient of spherical particles is provided in Equation 4 below.

(4) $$C_{d}=(\begin{array}{l} \frac{24}{R_{e}}(1+0.15R_{e}^{0.687}),R_{e}<1000\\ 0.44,R_{e}>1000 \end{array}$$

During the calculation process, since the gas flow density is approximately constant, a pressure solver is selected for the two-phase steady-state calculation. The pressure-velocity coupling adopts a simple algorithm (SIMPLE), the pressure is discretized using the second-order scheme, and the momentum and turbulence intensity are discretized using the second-order upwind scheme. Furthermore, when the particle volume fraction is below 10% - 12%, its effect on the airflow field can be neglected, and the single-phase coupling method is applied to solve the particle motion problem. By calculating the changes in momentum and mass of a single control volume in the model, the effect of airflow on the momentum and mass of the droplet particles can be determined.

#### 2.3.3 Atomizing nozzle model

**Figure 5:**
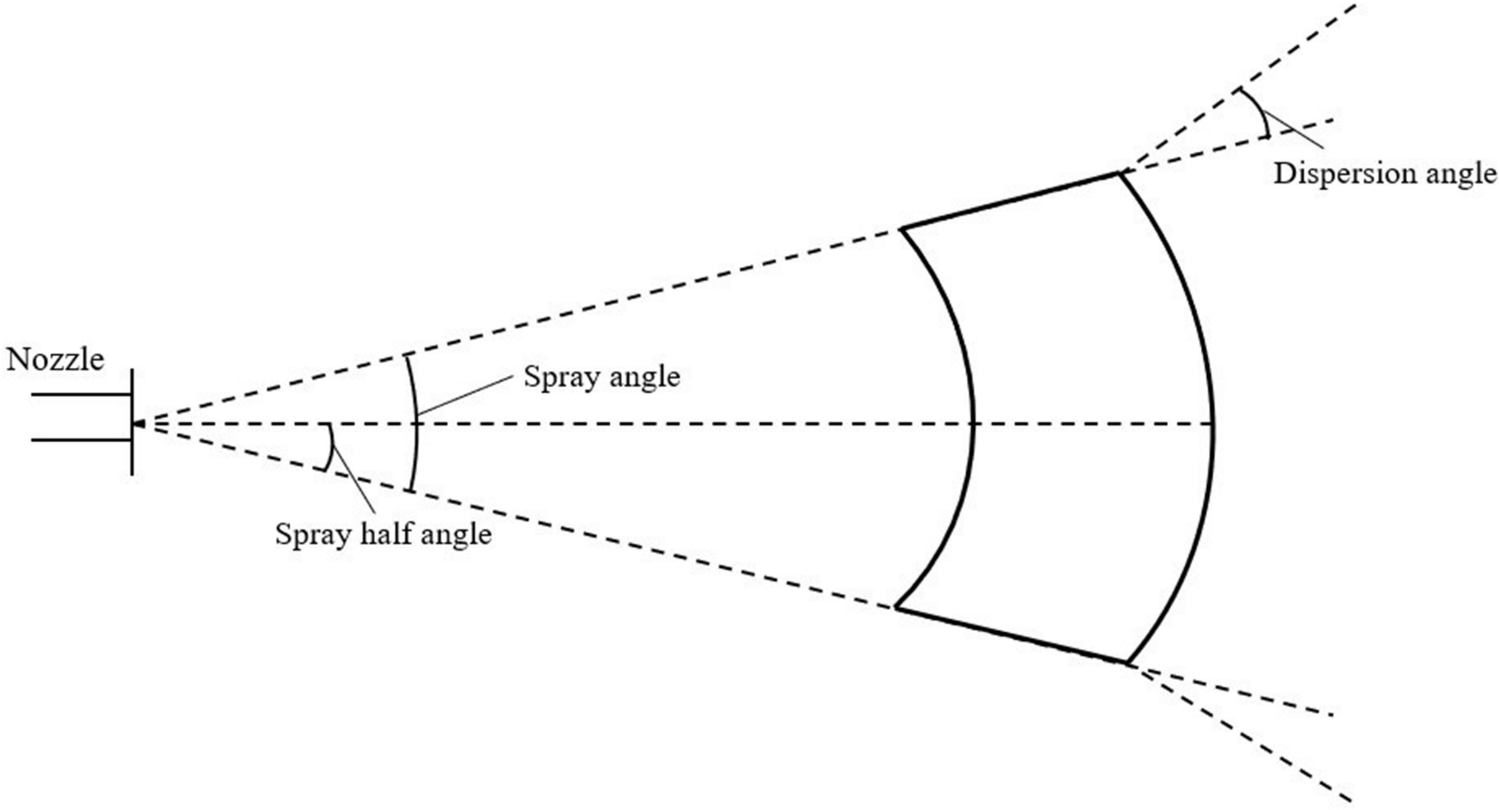
Schematic diagram of the flat fan atomizer model.

This study employs the flat fan atomizer within the Discrete Phase Model (DPM) framework to simulate the behavior of a fan atomizing nozzle. When a jet is formed through a flat fan-shaped nozzle, the resulting impact force is significant, leading to the formation of uniformly sized droplet particles. The spray range is broad and adjustable, making it widely applicable in orchard plant protection operations (Cao et al., 2020). This atomizing nozzle model is applied at the flat fan atomizer described earlier. Liquid flows out from here in the form of particles and enters the entire fluid calculation domain. **Figure 5** illustrates the schematic diagram of the fan-shaped nozzle, while the key model parameters are presented in **Table 1**.

**Table 1:** Key parameter settings of the flat fan atomizer model.

Name of parameters	Value
X-fan normal vector	0
Y-fan normal vector	0
Z-fan normal vector	1
Flow rate(kg/s)	a
Spray angle(deg)	b
Orifice width(mm)	c
Flat fan sheet constant	3
Dispersion angle(deg)	6

In the table, a, b, and c represent the values of flow rate, spray angle, and orifice width respectively.

#### 2.3.4 Droplet collision and breakup model

(1) Droplet collision model

The droplet collision model is applicable to collisions characterized by low Weber numbers, where the outcomes are limited to merging and rebounding (Jiang et al., 2022). Collisions among spray droplets occur exclusively within the same computational grid. When the positions of the internal spray droplets are uniformly distributed, the probability of capturing the droplets within the colliding body is:

(5) $$P_{1}=\frac{\pi {\left(r_{1}-r_{2}\right)}^{2}v_{rel}\Delta t}{V}$$

In Equation 5, *r_1_* and *r_2_* represent the radius of droplet 1 and droplet 2, *v_rel_* denotes the relative velocity between droplet 1 and droplet 2, *Δt* is the calculation time step, and *V* is the volume of the grid cell.

The probability distribution for the number of droplet collisions follows a Poisson distribution, as shown in Equation 6, *k* represents the number of droplet collisions.

(6) $$P(k)=\frac{\lambda^{k}e^{-\lambda }}{k!}$$

When two droplets collide, the collision result will also be determined as merging or rebounding, and the function (Sun et al., 2012) of its critical value is as shown in Equation 7.

(7) $$b_{crit}=(r_{1}+r_{2})\sqrt{\min(1.0,\frac{2.4f}{We})}$$

In Equation 8, *f* is a function of *r_1_*/*r_2_*, and its definition expression is:

(8) $$f(\frac{r_{1}}{r_{2}})=(\frac{r_{1}}{r_{2}})-2.4(\frac{r_{1}}{r_{2}})+2.7(\frac{r_{1}}{r_{2}})$$

The value of parameter *b*, obtained from the collision calculation, is $(r_{1}+r_{2})\surd Y$, where *Y* represents the average deviation. Droplet coalescence occurs when the impact parameter *b* is less than the value *b_crit_*; otherwise, they rebound.

(2) Droplet breakup model

Droplets experience shear forces in the airflow field. The aerodynamic forces acting on them may exceed the capacity of their surface tension, causing the droplets to deform and fragment into smaller particles. This process is characterized by the *We* number:

(9) $$W\text{e}=\frac{\rho_{\text{air}}v^{2}d}{\sigma }$$

In Equation 9, *We* represents the collision Weber number, *ρ_air_* represents the density of air, *v* represents the relative velocity, *d* represents the droplet diameter, and *σ* represents the surface tension coefficient.

Ansys Fluent software supports two droplet breakup models: the Taylor Analogy Breakup(TAB) model and the Wave Breakup model. The TAB model is primarily applied in spray processes with a low *We* number and is suitable for low-speed spray processes, while the wave fragmentation model is applicable when *We* > 100 and is typically used in high-speed spraying (Marek, 2013). In conclusion, since this study involves simulating the agricultural spray operation environment, which corresponds to a low-speed spray process, the TAB droplet breakage model is selected.

#### 2.3.5 Boundary conditions

During the numerical simulation of gas-liquid coupling, both initial and boundary conditions must be defined. This section focuses on the distribution of the external flow field of multiple air ducts during sprayer operation, where the gaseous phase is modeled as air at standard temperature, and the liquid phase is represented by water liquid, with the particle type specified as droplets in the Discrete Phase Model (DPM). Based on previous selections of the centrifugal fan and simulations of the internal flow field within the air delivery system, the average velocity for each air inlet and outlet was determined. The air inlets for all the air outlets were specified as velocity inlets, with a velocity of 45 m/s. Meanwhile, set the turbulence intensity to 5% and the turbulence viscosity ratio to 10. The gas-liquid two-phase flow model incorporates the DPM model, building on the single-phase airflow field model. The boundary condition for the wall at the end of the computational domain is specified as a trap, with the liquid film wall option simultaneously activated. The remaining outlet boundaries and the ground wall are set as escape boundaries, where droplet particles are allowed to exit. The walls of the four air outlets are designated as reflective boundaries, meaning that particles will be reflected upon contact.

### 2.4 Simulation test on influencing factors of multi-duct spray operation

**Figure 6:**
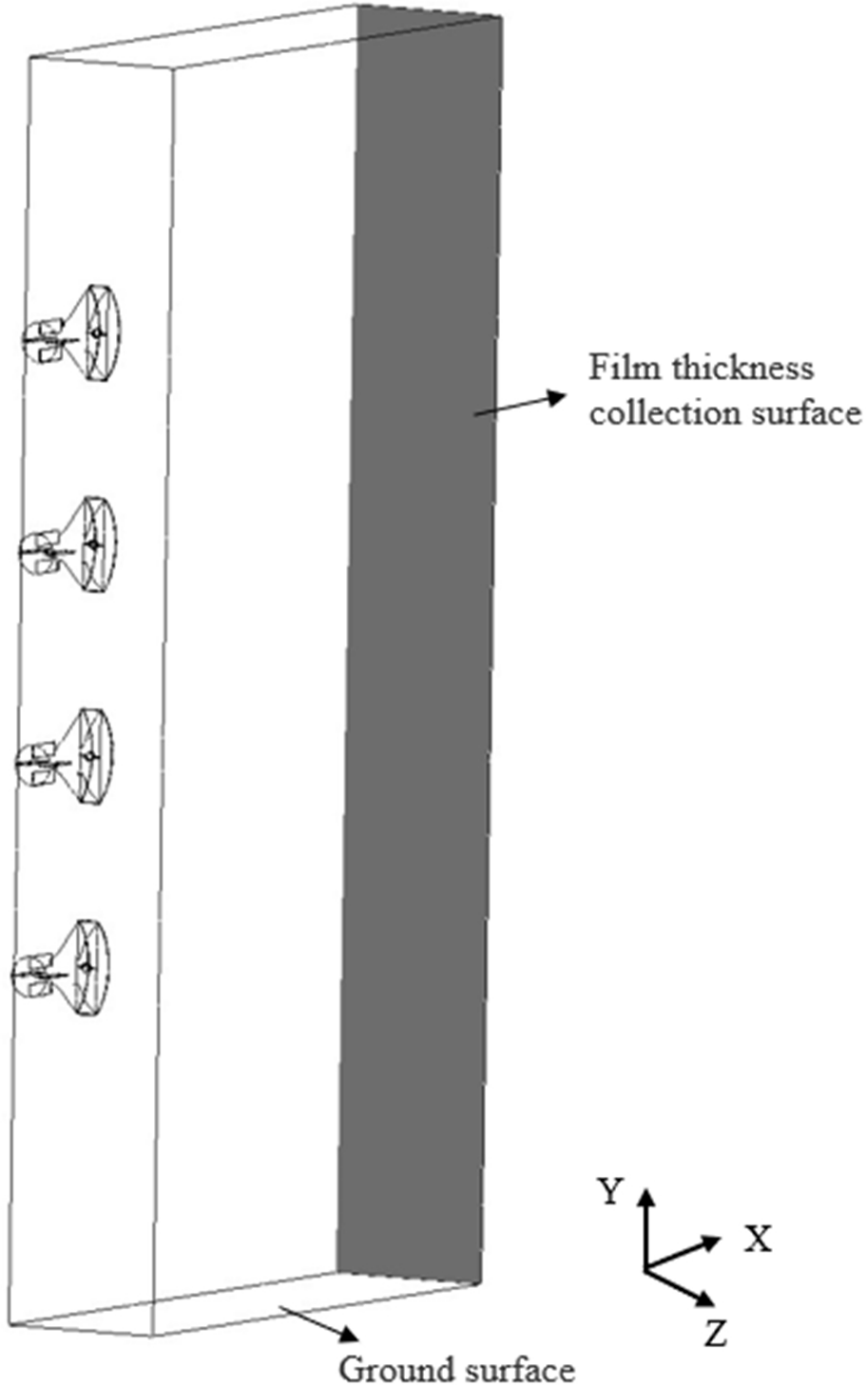
Schematic diagram of the liquid film thickness collection surface.

The Euler wall liquid film model presents an innovative approach for simulating the formation and evolution of wall liquid films. It allows for a more accurate calculation of the adhesion and evolution processes of liquid films on the wall, providing a more realistic representation of the formation of wall liquid films by water droplet particles. Additionally, the thickness of the liquid film serves as an indirect indicator of droplet deposition. When the spraying simulation time remains constant, a thicker liquid film correlates with a higher aggregation of droplets. This indicates that the effectiveness of droplet deposition improves as the liquid film thickness increases (Chen et al., 2015). Thus, the liquid film thickness at the end plane of the computational domain is utilized as one of the evaluation metrics for the simulation experiments, as illustrated in **Figure 6**. Moreover, in the post-processing of Ansys Fluent, the uniformity index is used to evaluate the uniformity of the liquid film thickness distribution on the liquid film collection surface, with values ranging from 0 to 1. A higher value indicates greater uniformity. Therefore, to indirectly assess the uniformity of droplet deposition, this study incorporates the uniformity index of the liquid film distribution as another evaluation metric for the experimental results, with the simulation outcomes being jointly evaluated using both indices. Additionally, this study reasonably selects the experimental factors and determines the range of factor levels through pre-simulation experiments.

The liquid flow rate at the nozzle outlet quantifies the mass of liquid discharged from the nozzle per unit of time, serving as a critical parameter influencing the extent of droplet deposition. Furthermore, the flat fan atomizer employed in this study allows for modulation of the spray angle, thereby increasing or decreasing the spray amplitude at the nozzle outlet. The magnitude of the spray amplitude influences droplet interactions between sprays from multiple nozzles, ultimately affecting the final droplet deposition pattern. The nozzle orifice width directly determines the droplet particle outlet diameter. The droplet particle outlet diameter influences the extent of droplet breakage during interactions and collisions, thereby affecting the atomization efficiency. Additionally, it exerts a significant influence on the droplet deposition outcome.

In this study, the outlet flow rate, spray angle, and orifice width of the nozzle are chosen as the primary test factors. The levels for each factor are carefully selected to ensure a meaningful investigation. To optimize the simulation process and reduce the computational scale, only a subset of these levels is chosen for the simulation experiments. The corresponding test scheme, as summarized in **Table 2**, outlines the specific combinations of these factors for the simulations.

**Table 2:** Simulation test scheme table.

Test groups	Flow rate(kg/s)	Spray angle(deg)	Orifice width(mm)
A B C D	0.03 0.04 0.05 0.06	60 60 60 60	0.6 0.6 0.6 0.6
E F G H	0.03 0.03 0.03 0.03	50 60 70 80	0.6 0.6 0.6 0.6
I J K L	0.03 0.03 0.03 0.03	70 70 70 70	0.4 0.6 0.8 1.0

### 2.5 Simulation test on the influence of the air outlet opening degree and interval on the characteristics of multi-duct airflow field

**Figure 7:**
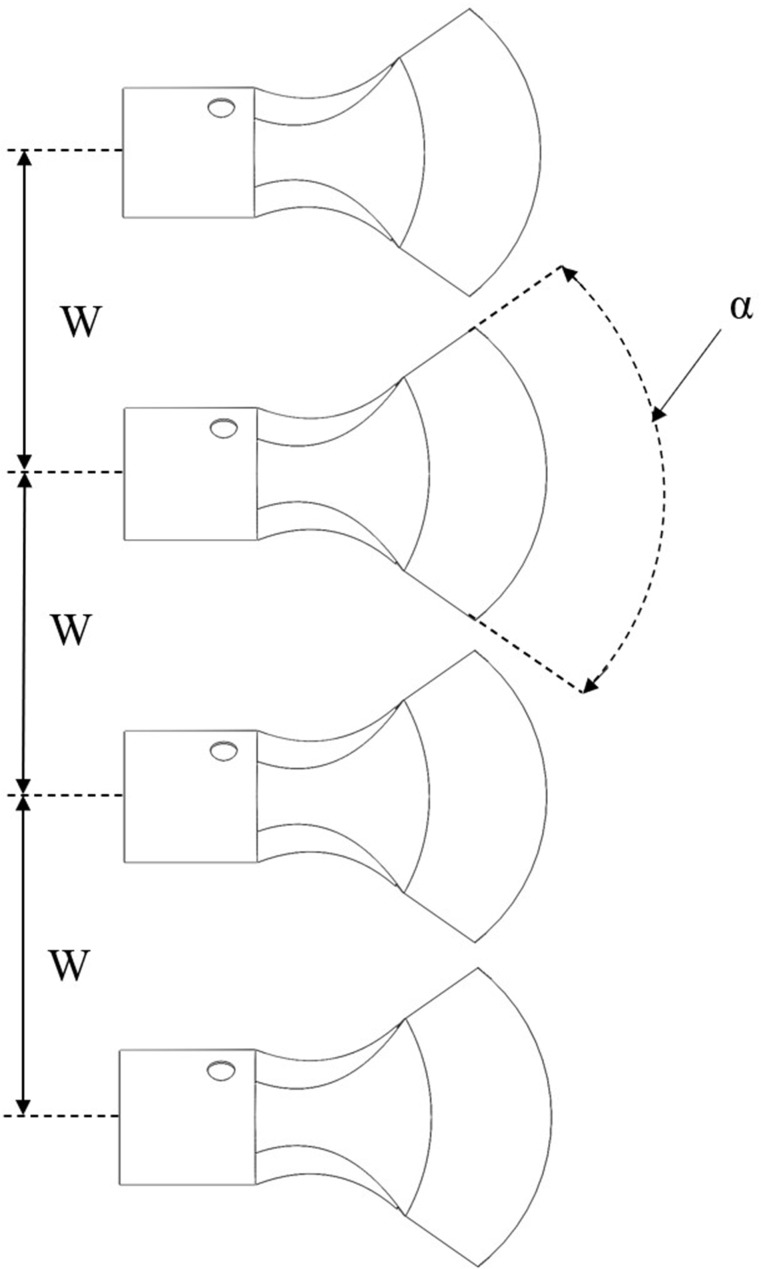
Schematic diagram of the opening degree and interval of the air outlet.

The degree of opening of the air outlet and its interval configuration significantly influences the distribution of the multi-duct spray, as illustrated in **Figure 7**. In the figure, *α* denotes the degree of opening of the air outlet, and *W* represents the interval between the air outlets. This study investigates the effect of varying test levels of air outlet opening degrees and interval on the effective working height of the airflow field, using CFD simulations. However, the simulation content related to the spray was not covered, so the DPM simulation model was not included in the simulation process of this section. Furthermore, monitoring was implemented at the boundary of the computational domain to assess the uniformity of the velocity distribution within the airflow field and to investigate how the air outlet opening degree and interval affect this uniformity. This test provides valuable data and insights that can support the future optimization of the multi-duct sprayer for profiling spray operations.

If the opening of the air outlet is excessively large, it may result in the loss of its air-guiding function. Additionally, the spacing between the air outlets influences the interaction among them. Accordingly, the levels of the test factors are carefully selected in this study. The design of the simulation test scheme is outlined in **Table 3**.

**Table 3:** Simulation test scheme table.

Test groups	The air outlet opening degree *α*(deg)	The air outlet interval *W*(mm)
I II III IV V VI VII VIII IX	70 80 90 70 80 90 70 80 90	500 500 500 600 600 600 700 700 700

### 2.6 Simulation test of atomization characteristics of multi-duct flow field under the action of airflow field

When spray droplets traverse the external flow field generated by multiple air ducts, phenomena such as droplet breakage, collision, and coalescence occur due to the influence of the airflow field. This study aims to investigate the variation of droplet size under the influence of the airflow field generated by multiple air ducts, providing simulation data as a reference for the rational selection of the optimal distance between the multi-air duct sprayer and the fruit tree canopy to achieve precision spraying. Previous studies have simulated the internal flow field of the multi-duct sprayer air distribution system. Based on previous research data, it can be concluded that when the centrifugal fan operates at rated speed, the average inlet velocity at the air outlet is approximately 45 m/s. In this test, three air velocity levels were chosen for simulation, with 35 m/s, 40 m/s, and 45 m/s corresponding to schemes A, B, and C, respectively. Meanwhile, while maintaining other parameters constant, the air outlet opening degree is set at 70°, the interval is 500 mm, the outlet flow rate of the flat fan atomizer is 0.03 kg/s, the spray angle is 70°, and the orifice width is 0.6 mm.

**Figure 8:**
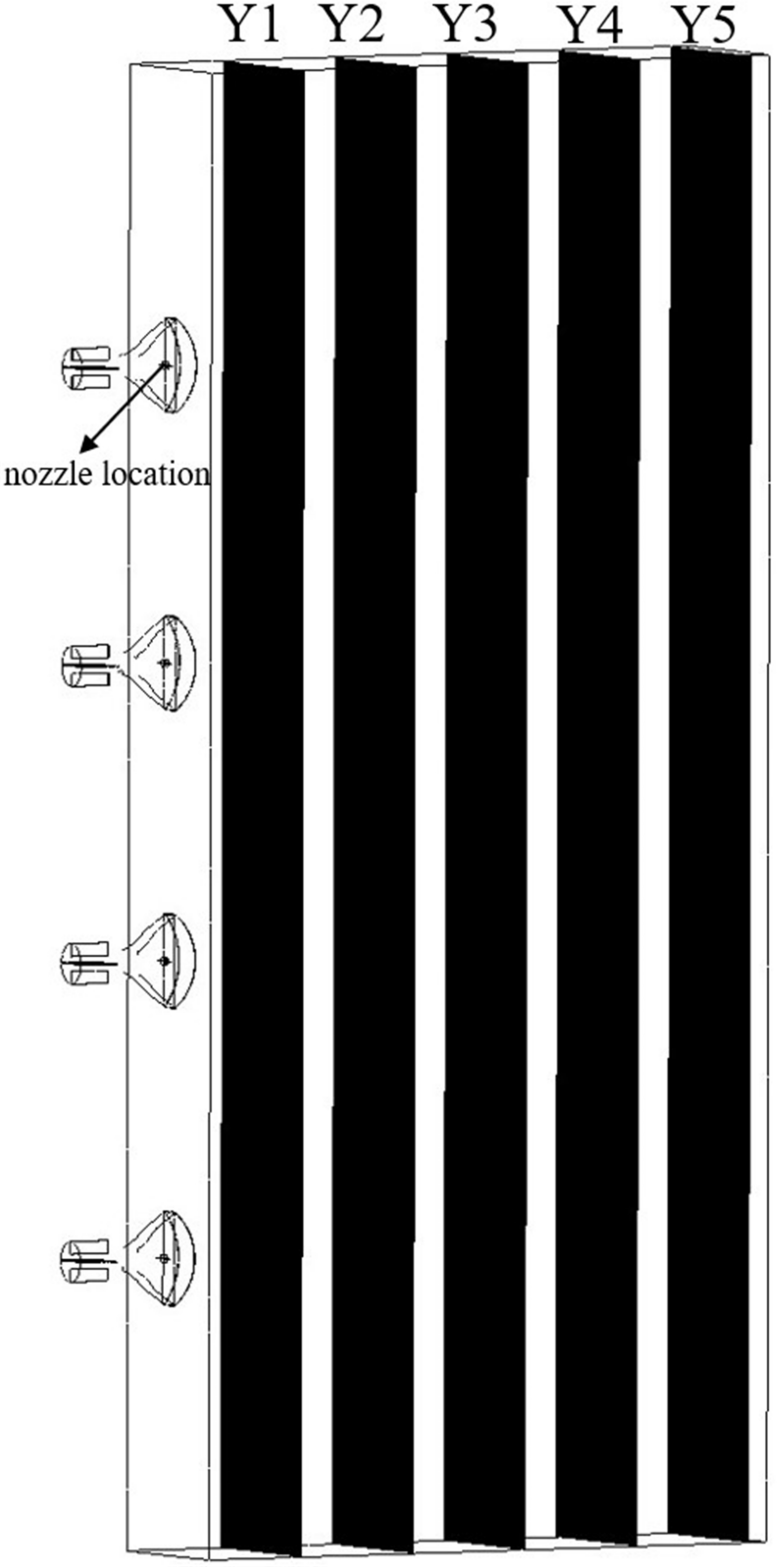
Schematic diagram of the monitoring planes for particle diameter distribution.

Monitoring planes were set at distances of 0.3 m, 0.6 m, 0.9 m, 1.2 m, and 1.5 m from the nozzle location, denoted as Y1, Y2, Y3, Y4, and Y5, respectively, as illustrated in **Figure 8**. Subsequently, the temporal variations in droplet size on the monitoring surfaces are analyzed to derive the governing principles of droplet size distribution within the airflow field, followed by the atomization behavior. Finally, within the DPM model, the droplet collision, breakup, and merging models are activated, and the species transport equation is concurrently enabled for computational resolution.

### 2.7 Droplet particle size measurement test

To verify the CFD simulation model established in this study and enhance its credibility in practical applications, this section presents a droplet particle size measurement test conducted in the agricultural engineering building of South China Agricultural University.

The test was conducted using the HNB-PW1000 laser particle size analyzer. The measurement range of the HNB-PW1000 laser particle size analyzer spans from 1 μm to 1000 μm. Both the measurement accuracy error and the repeatability error are less than 1%. Its working principle involves determining the particle size distribution by measuring the angle and intensity of the scattered light produced by the interaction of the particles with the laser. Additionally, due to the excellent monochromaticity and strong directionality of the laser, the analyzer achieves high precision and accuracy in particle measurement.

**Figure 9:**
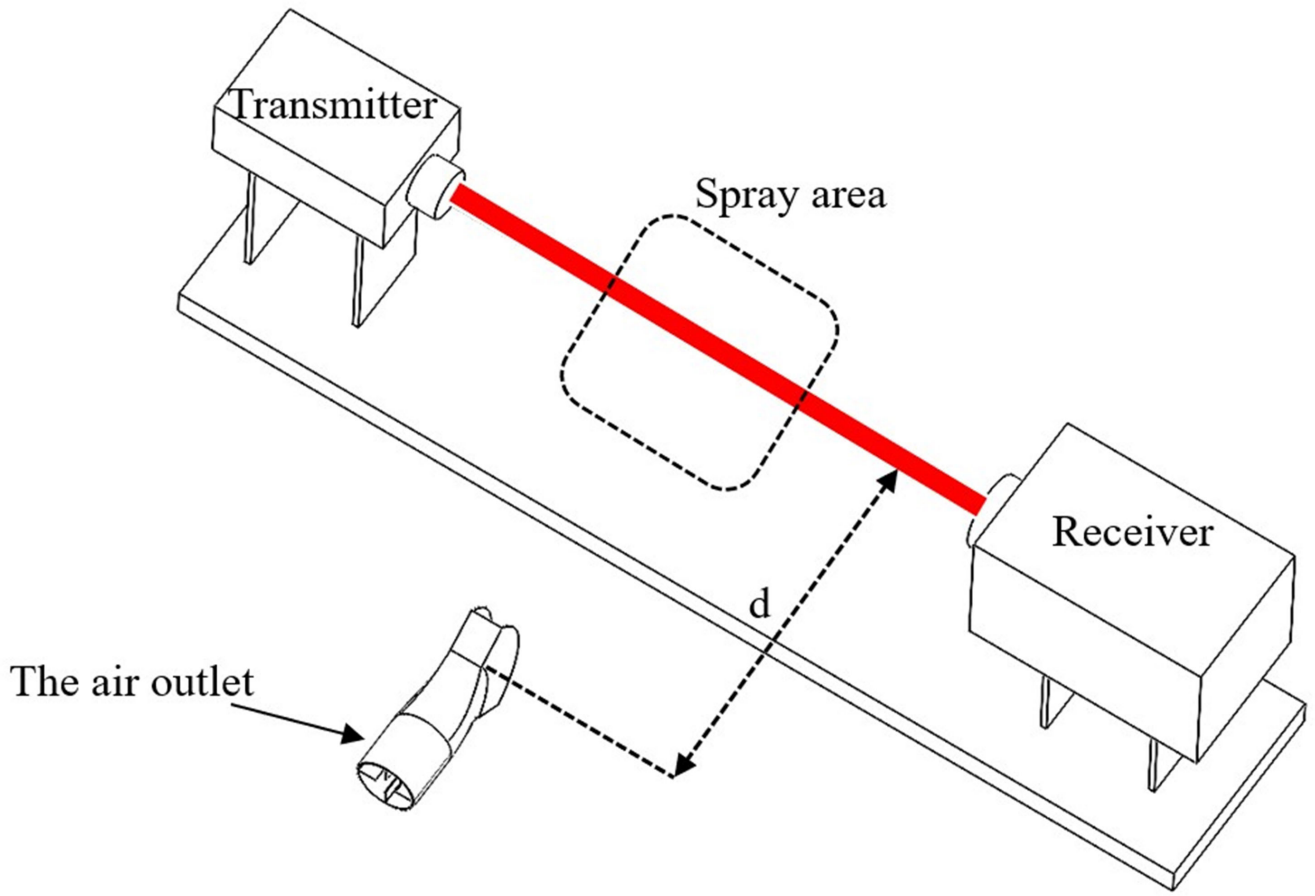
Schematic diagram of droplet particle size measurement test.

Due to the substantial dimensions of the multi-duct sprayer developed in this study, it is not feasible to assess the entire spray system. Consequently, this test was limited to testing a single duct of the multi-duct sprayer. A flat fan atomizer with a 70° spray angle and a 0.6 mm orifice width was selected for this test. The flow rate was set to 0.03 kg/s. Simultaneously, an air outlet with a 70° opening angle was selected. The rotational speed of the centrifugal fan was calibrated to 961.7 r/min, the rated speed of the fan, corresponding to an inlet air velocity of 45 m/s at the air outlet. Throughout the test, the laser particle size analyzer was positioned at a distance *d* from the outlet, with *d* taking values of 0.3 m, 0.6 m, 0.9 m, 1.2 m, and 1.5 m for five test groups. Each test group was repeated three times to record the measured droplet particle sizes. The particle size is calculated using the volume mean diameter (VMD) of the measurement values. The average of the droplet particle size measurements was then calculated as the test value. The droplet particle size measurement test schematic diagram is shown in **Figure 9**.

## 3 Results and discussion

### 3.1 Simulation test results and analysis of influencing factors in multi-duct spray operations

**Figure 10:**
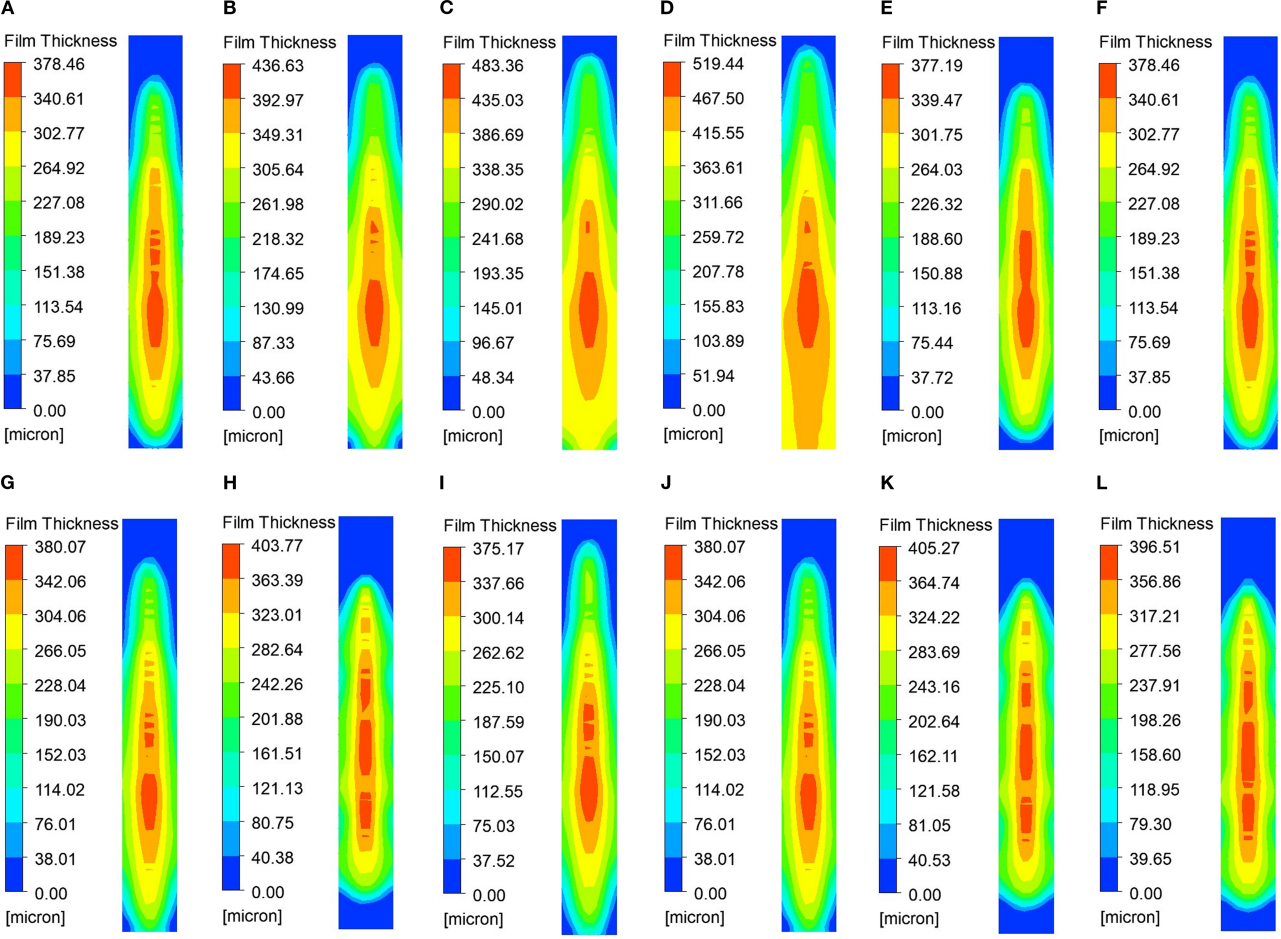
Contours of liquid film thickness for the considered cases: **(A)** 0.03,60,0.6; **(B)** 0.04,60,0.6; **(C)** 0.05,60,0.6; **(D)** 0.06,60,0.6; **(E)** 0.03,50,0.6; **(F)** 0.03,60,0.6; **(G)** 0.03,70,0.6; **(H)** 0.03,80,0.6; **(I)** 0.03,70,0.4; **(J)** 0.03,70,0.6; **(K)** 0.03,70,0.8; **(L)** 0.03,70,1.0.

The simulation results are summarized in **Table 4**, Additionally, the simulation results for each group are depicted in **Figures 10A–L**. The figure indicates that the regions of highest concentration of liquid film thickness are primarily located in the middle and lower sections of the collection surface. This outcome is attributed to the consideration of gravitational effects on droplet movement in the simulation process described in this study (Chen et al., 2020). From the overall analysis of **Table 4** and **Figures 10A–L**, it is evident that the flow rate has the most substantial effect on the mean liquid film thickness, whereas the spray angle and nozzle orifice width show no significant impact on the liquid film thickness but notably influence the uniformity index of the liquid film distribution.

**Table 4:** Simulation results at different test levels.

Test groups	Mean value of liquid film thickness(μm)	Uniformity index
A B C D	197.31 252.81 301.17 340.71	0.7521 0.8032 0.8325 0.8465
E F G H	196.67 197.31 197.28 196.37	0.7466 0.7521 0.7550 0.7068
I J K L	197.42 197.28 196.83 196.72	0.7663 0.7550 0.7073 0.7090

(1) The influence of flow rate on the thickness and uniformity of the liquid film

As shown in **Table 4** and **Figures 10A**–**10D**, an increase in flow rate leads to a significant rise in the mean liquid film thickness, from 197.31 μm to 340.71 μm, suggesting that flow velocity is the primary factor driving this increase. Concurrently, the uniformity index increased from 0.7521 to 0.8465, indicating that a higher flow rate promotes a more uniform distribution of the liquid film. This can be attributed to the fact that an increased flow rate reduces droplet aggregation.

(2) The influence of spray angle on the thickness and uniformity of the liquid film

As shown in **Table 4** and **Figures 10E**–**10H**, the change in spray angle has a minimal effect on the liquid film thickness, with an average fluctuation of less than 1 μm, suggesting that the spray angle has little sensitivity to the film thickness. However, the uniformity index initially increases and then decreases as the spray angle increases. For group H, which uses a large spray angle, the uniformity index is the lowest at 0.7068, indicating that an excessively large spray angle may result in uneven droplet dispersion.

(3) The influence of orifice width on the thickness and uniformity of the liquid film

The analysis of **Table 4** and **Figures 10I**–**10L** reveals that the nozzle orifice width has a relatively minor impact on the thickness of the liquid film, with the fluctuation of the mean value remaining below 1μm. This suggests that the orifice width does not significantly affect the liquid film thickness. The uniformity index achieves its highest value of 0.7663 when the inner diameter is at its minimum (Group I). Conversely, when the inner diameter is 0.8 mm (Group K), the uniformity index decreases markedly. This suggests that a smaller orifice width enhances the uniformity of the liquid film. This is attributed to the fact that a smaller orifice width produces finer droplets, resulting in a more uniform distribution.

Based on the above analysis, **Table 5** is summarized as follows:

**Table 5:** Comprehensive analysis and optimization suggestions.

Test factors	The influence of liquid film thickness	Influence on uniformity	Suggestion on optimization
Flow rate (0.03-0.06 kg/s)	Progressively increase	Progressively increase	The flow rate can be appropriately increased
Spray angle (50–80 deg)	Basically unchanged	First increase and then decrease	Choose a medium spray angle
Orifice width (0.4-1.0 mm)	Basically unchanged	Continuously decrease	Choose a smaller orifice width

As illustrated in **Table 5**, when selecting operational parameters, priority should be given to the flow rate, as it has an effective impact on enhancing the droplet deposition efficiency. Simultaneously, a moderate spray angle of approximately 70 degrees should be selected to prevent a large angle, which would result in a continuous decline in uniformity. Furthermore, nozzles with smaller orifice width should be employed to optimize the uniformity index.

### 3.2 Simulation test results and analysis of the influence of the air outlet opening degree and interval on the characteristics of multi-duct airflow field

**Figure 11:**
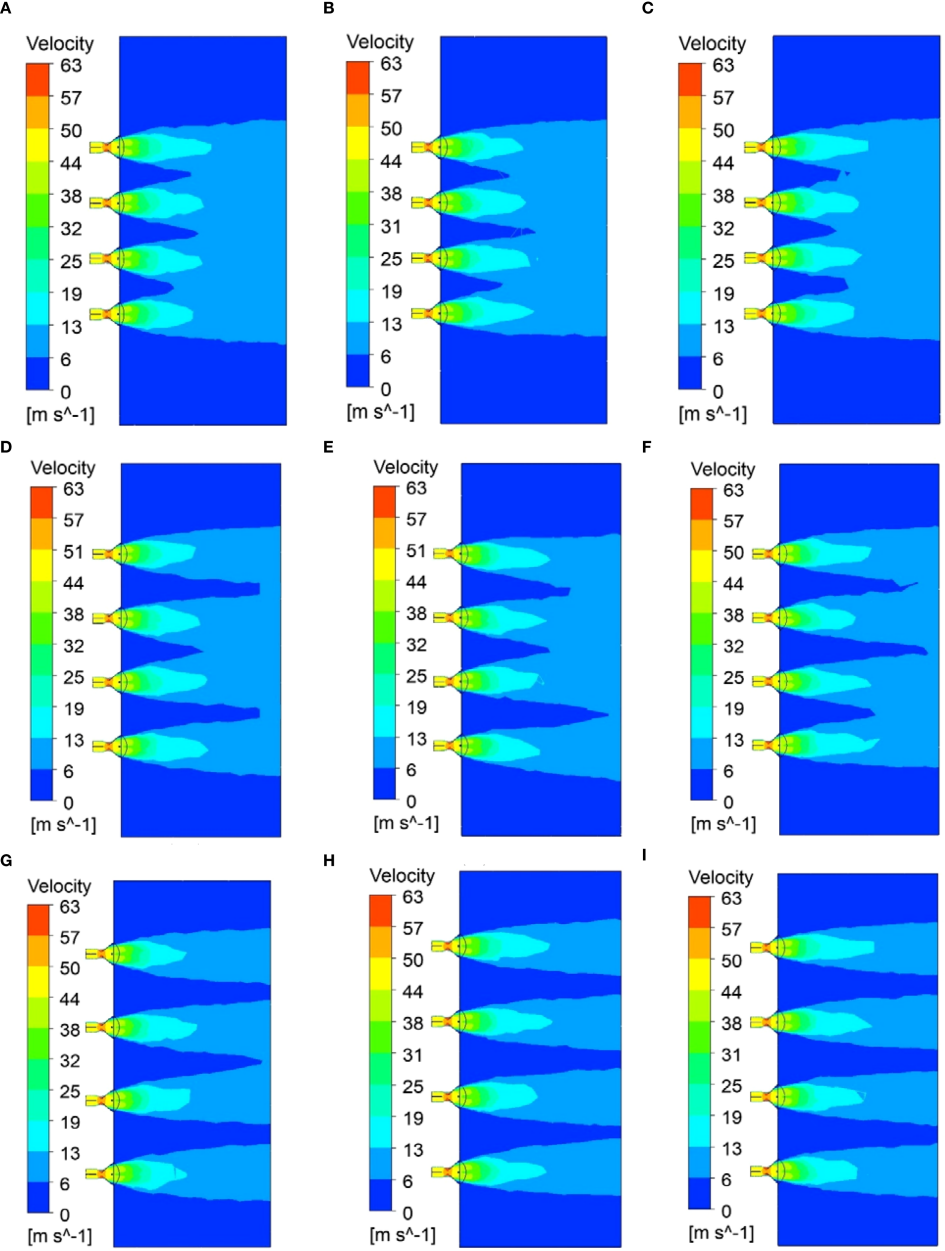
Simulation results at each test level: **(A)** 70,500; **(B)** 80,500; **(C)** 90,500; **(D)** 70,600; (E) 80,600; **(F)** 90,600; **(G)** 70,700; **(H)** 80,700; **(I)** 90,700.

The simulation results are summarized in **Table 6**, while the results for each test level are presented in **Figures 11A–I**. A separate analysis of groups “A, B, C”, “D, E, F”, and “G, H, I” shows that when the air outlet opening increases from 70° to 80°, the effective working height of the airflow field increases by 0.2 m. However, when the air outlet opening increases from 80° to 90°, the effective working height of the airflow field increases by 0.1 m. Separate analysis of groups “A, D, G”, “B, E, H”, and “C, F, I” shows that when the air outlet interval increases by 100mm, the effective operating height of the airflow field increases by 0.3 m. Additionally, as illustrated in **Table 6**, when the outlet opening degree and interval are increased, the uniformity index of the airflow velocity distribution at the domain’s end exhibits a downward trend. This occurs because as the air outlet opening and interval increase, the interaction strength at each air outlet weakens, and the areas of high-speed airflow where local interactions occur decrease. However, it is important to note that when the interval between the air outlets is 700 mm, the airflow field prevents the airflow from each air outlet from converging, which may influence the transport and deposition of the droplets.

**Table 6:** Simulation results at different test levels.

Test groups	Effective working height (m)	Uniformity index
A B C	3.6 3.8 3.9	0.5955 0.5489 0.5221
D E F	3.9 4.1 4.2	0.6461 0.6013 0.5854
G H I	4.2 4.4 4.5	0.6929 0.6741 0.6693

### 3.3 Simulation test results and analysis of atomization characteristics of multi-duct flow field under the action of airflow field

**Figure 12:**
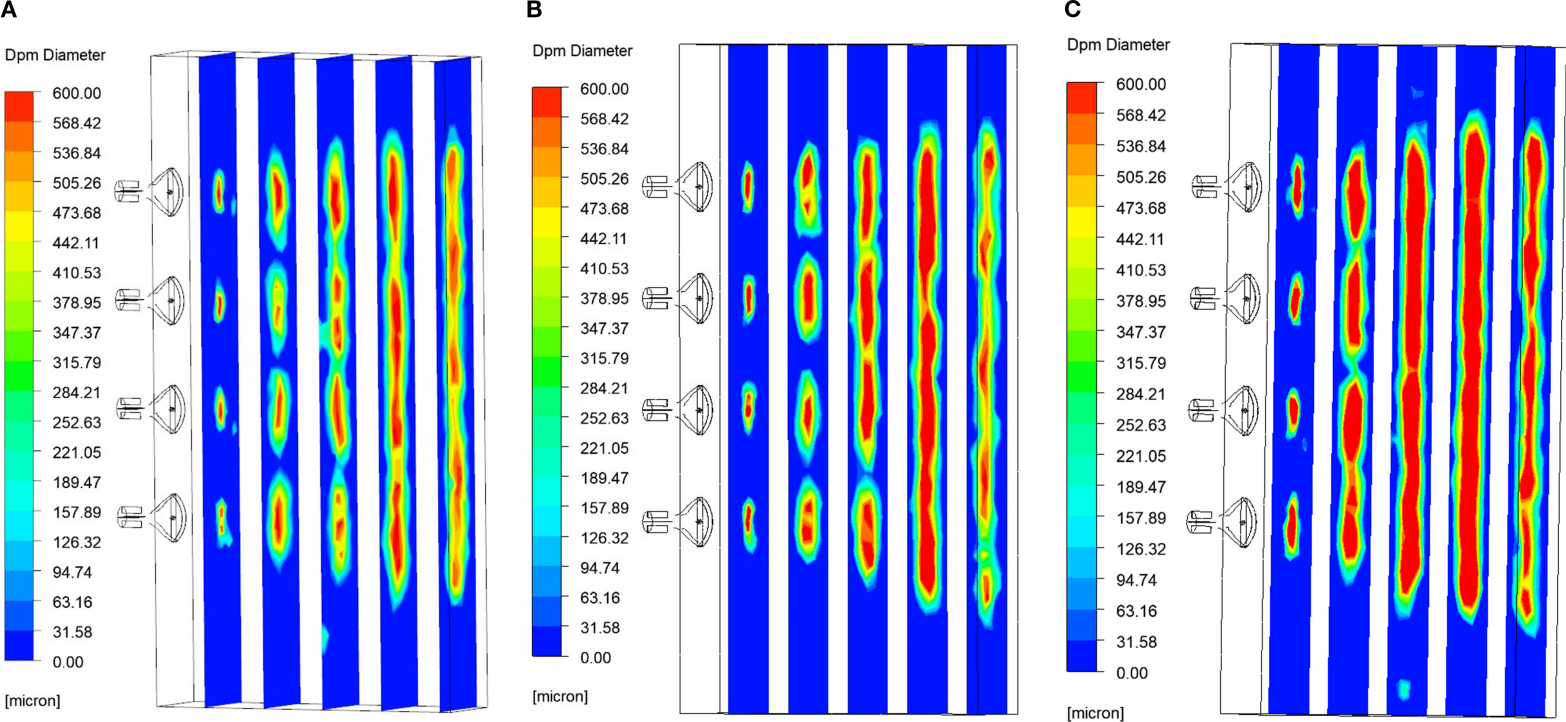
Contours of particle size distribution of droplets under the influence of airflow field for three considered values of air velocity: **(A)** air velocity 45 m/s; **(B)** air velocity 40 m/s; **(C)** air velocity 35 m/s.

**Figures 12A–C** illustrates the contours of the droplet size distribution as influenced by the airflow field at three different air velocity. Additionally, **Figures 12A–C** summarizes the average droplet size for each monitoring surface.

**Figure 13:**
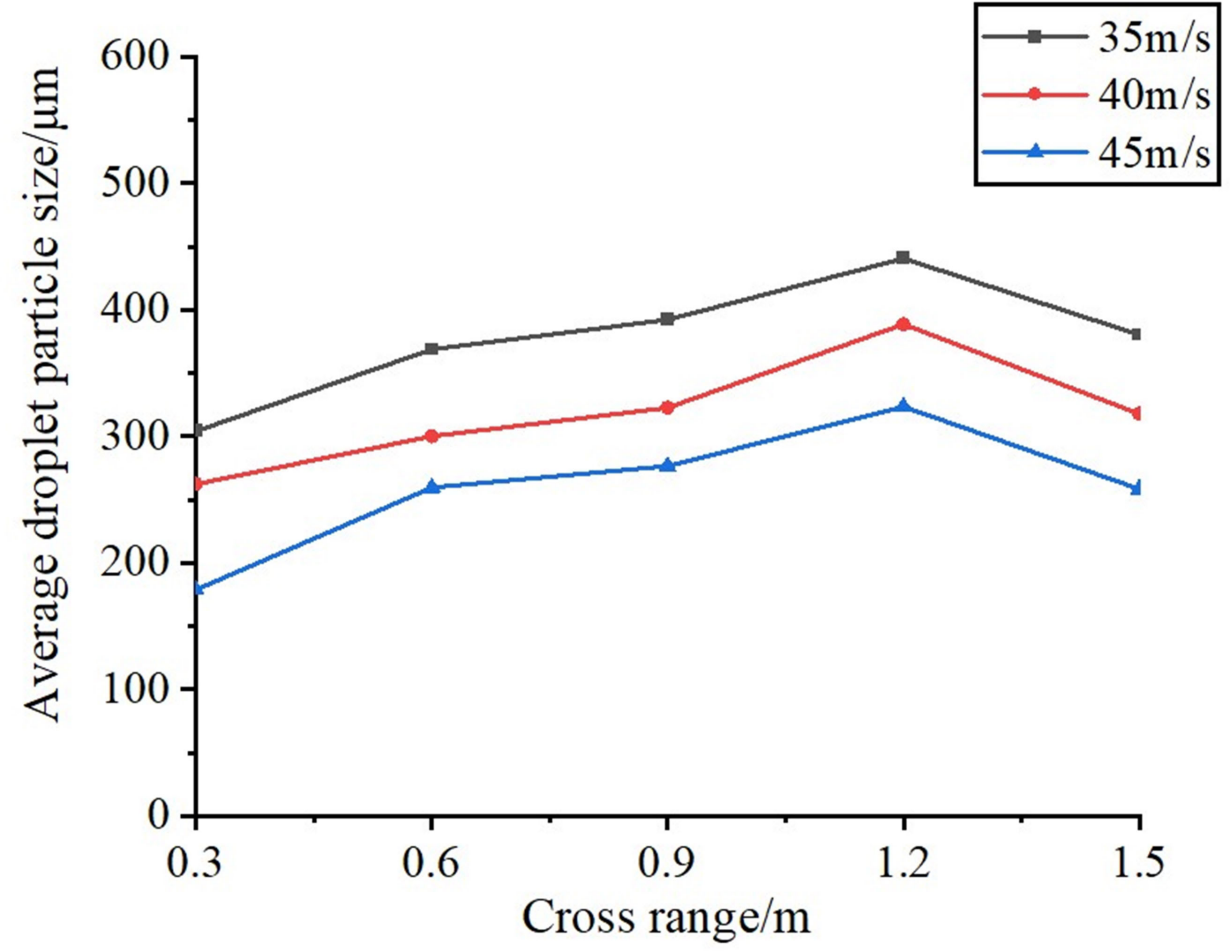
Particle size distribution curves of each detection surface for three considered values of air velocity.

As shown in **Figures 12A–C**, **13**, when the liquid is ejected from the nozzle, the initial atomization occurs as a result of the interaction between jet instability (Mu et al., 2020) and the airflow field, leading to a significant reduction in droplet size. Subsequently, the smaller droplets collide and coalesce in the airflow field due to their instability, continuing until they reach approximately 1.2 meters from the air outlet. Once the droplet size reaches a certain threshold and growth ceases, although the wind speed has attenuated to some extent, it still exerts sufficient shear force to induce a second atomization, resulting in a further reduction in droplet size, which enhances droplet deposition.

As illustrated in **Figure 13**, the particle size of the droplets during the first atomization increases as the initial wind speed decreases, specifically at air velocity of 45 m/s, 40 m/s, and 35 m/s. This phenomenon occurs because a reduction in wind speed leads to a corresponding decrease in the shear force exerted on the droplets, thereby diminishing the degree of atomization. Furthermore, as the wind speed in the field decays with increasing lateral distance, a larger initial wind speed results in a smaller maximum critical particle size achievable through droplet merging. Additionally, as the droplets travel approximately 1.2 meters from the air outlet and undergo the second atomization, their particle size also decreases.

As demonstrated in the previous discussion, the atomization characteristics of spray droplets are notably influenced by the airflow field as they travel through the multi-channel wind environment. The findings of this study offer valuable insights for the subsequent selection of an optimal operational distance between the sprayer rows. By appropriately selecting the operational distance, an optimal droplet size can be achieved to suit the operational requirements of various orchards.

### 3.4 Results and analysis of droplet particle size measurement test

The results of the tests are summarized in **Table 7**. Analysis of **Table 7** reveals that the test values of droplet particle size initially increase and then decrease as the d value increases, a trend that aligns with the simulation values shown in **Figure 13**. Concurrently, the average of the three measurement values in each test group was computed, and the relative error between these values and the simulation values was calculated. The relative errors between the test values and the simulation values across the five test groups ranged from 11.4% to 15.3%, all of which were below 16%, thereby confirming the reliability and applicability of the CFD simulation model established in this study.

**Table 7:** The results of droplet particle size measurement test.

d(m)	Measurement values of droplet particle size (μm)	Test values(μm)	The relative error compared to the simulation values
0.3	205.96 197.28 202.58	201.94	13.1%
0.6	291.12 319.16 287.35	299.21	15.3%
0.9	309.41 315.58 312.97	312.32	13.3%
1.2	362.32 355.74 363.44	360.50	11.5%
1.5	288.63 296.52 290.19	291.78	11.4%

## 4 Conclusions

The traditional prototype testing method is time-consuming and incurs high costs. Utilizing CFD method, this study developed a fluid simulation model for the multi-duct sprayer and conducted a series of simultaneous gas-liquid coupling simulations to investigate the effects of various factors on the multi-duct spray flow field. The primary conclusions drawn from this study are as follows:

(1) The impact of spray flow rate, spray angle, and nozzle orifice width on droplet deposition rate and its uniformity was investigated through a simulation test examining the factors influencing multi-air duct air-delivery spray operations. During the simulation experiment, the thickness of the liquid film and its distribution uniformity were assessed indirectly. The simulation results demonstrate that as the nozzle flow rate increases from 0.03 kg/s to 0.06 kg/s, the average thickness of the liquid film rises from 197.31 μm to 340.71 μm, and the uniformity index increases from 0.7521 to 0.8465. This suggests that higher flow rates effectively enhance droplet deposition and improve distribution uniformity. A medium spray angle (70°) yields the highest uniformity index for the liquid film. In contrast, an excessively large angle results in a significant reduction in uniformity. The nozzle with a smaller orifice width increases the uniformity index to 0.7663 by generating finer droplets, outperforming the nozzle with a larger orifice width. However, it is important to note that excessively small droplet sizes increase the risk of droplet drift. The influence of this operational factor provides valuable insights for the further optimization of operational parameters for the multi-duct sprayer in orchard applications.

(2) This study conducted simulation tests to investigate the effects of the opening degree and interval of the air outlets on spray operation in multi-ducts. The results revealed the impact of these factors on both the effective working height and the uniformity of the velocity distribution at the end of computational domain of the airflow field. As the outlet opening degree increases from 70° to 80°, the effective operational height of the airflow field increases by 0.2 m, and a further increase from 80° to 90° results in an additional increase of 0.1 m. Moreover, for every 100 mm increase in the outlet spacing, the effective working height of the airflow field rises by 0.3 m. Furthermore, the results also show that as both the outlet opening degree and spacing increase, the uniformity of the velocity distribution at the end of computational domain of the airflow field decreases to varying extents. These findings may serve as a reference for optimizing the profiling spray operations of multi-duct sprayers.

(3) Simulation tests were conducted to examine the atomization characteristics of droplets in the multi-channel flow field influenced by the airflow field. Upon ejection from the nozzle, the droplets undergo initial atomization. Subsequently, due to a rapid reduction in particle size, the smaller droplets continue to travel through the airflow field, where they collide and merge with larger droplets, increasing their size. Upon reaching approximately 1.2 meters from the air outlet, the droplets undergo secondary atomization due to the sheer force of the airflow field, resulting in a further reduction in droplet size, which facilitates deposition within the fruit tree canopy. This phenomenon provides valuable insights for the subsequent optimization of row spacing in orchard operations using multi-duct sprayers.

(4) The reliability of the CFD simulation model presented in this study was validated by conducting droplet particle size measurement test. When all other conditions were maintained consistent with the simulation parameters, and the initial air velocity at the air outlet was 45 m/s, the trends of the test and simulation values were similar, with the relative error ranging from 11.4% to 15.3%, which falls within an acceptable range. This confirms the reliability of the CFD simulation model presented in this study and its credibility for practical applications.

The conclusions of this study can serve as a reference for further optimization and enhancement of the operational efficiency of the multi-duct sprayer in orchards. Additionally, it can provide data support for the subsequent transformation of conventional pesticide application practices associated with this sprayer.

## Data Availability

The raw data supporting the conclusions of this article will be made available by the authors, without undue reservation.
